# Use of mHealth interventions to enhance self-regulation and self-efficacy in individuals living with obesity: Scoping review

**DOI:** 10.1177/20552076261474266

**Published:** 2026-08-19

**Authors:** Marcela Ely, Asghar Ahmadi, Syed M. Shariq, Andrea Duchonova, Roman Filo, Barbora Kastovska, Ondrej Zach, Holly B. Jimison, Steriani Elavsky

**Affiliations:** 1 Department of Human Movement Studies, 562307 Ostrava University, Ostrava, Czech Republic; 2 Faculty of Physical Education and Sport, 156922 Charles University, Prague, Czech Republic; 3 Khoury College of Computer Sciences, 205735 Northeastern University, Boston, MA, USA

**Keywords:** behavior change techniques, just-in-time adaptive interventions, ecological momentary assessment, weight management, self-regulation, self-efficacy

## Abstract

**Objectives:**

Mobile health (mHealth) technologies are increasingly employed to facilitate lifestyle modification and weight management among individuals with obesity. However, their effectiveness depends on the integration of theory-driven design. Within Social Cognitive Theory, self-regulation and self-efficacy are considered key mechanisms for initiating and sustaining health behavior change. This scoping review aimed to map and categorize mHealth intervention components related to self-regulation and self-efficacy, explore their digital operationalization and personalization strategies, and identify the theoretical frameworks guiding their design.

**Methods:**

A systematic literature search was conducted across six databases to identify studies that examined mHealth interventions targeting adults living with obesity and addressing behavioral modification in obesity management. The review applied Joanna Briggs Institute (JBI) methodology for scoping reviews and is reported following the PRISMA-ScR guidelines.

**Results:**

Forty-four studies met the inclusion criteria. Theoretical grounding was present in a total of twenty-five interventions (25/44; 56.8%), with Social Cognitive Theory being the most prevalent framework (15/25; 60%). Findings indicate widespread integration of self-regulation and self-efficacy components within mHealth interventions, operationalized through in-app logs, wearable devices, prompts and reminders, gamification, and social support etc. While mHealth technologies have increasingly integrated advanced sensors and AI-enabled features, the aspect of personalization has remained predominantly static rather than adaptive. The mHealth interventions aimed at managing obesity commonly incorporate components related to self-regulation and self-efficacy, yet theoretical integration, dynamic personalization, and consistent reporting remain limited.

**Conclusion:**

This scoping review highlights the need for clearer documentation of behavioral mechanisms and proposes reporting recommendations to improve transparency and replicability.

Obesity presents a significant public health challenge, imposing a considerable economic burden and resulting in adverse health outcomes.^1,2^ Characterized by the excessive accumulation of body fat, obesity can detrimentally affect health and well-being.^1^ It is a complex chronic condition influenced by genetic, environmental, behavioral, and socioeconomic factors.^3^ According to the World Health Organization, in 2022, the number of adults exceeding a healthy weight reached 2.5 billion worldwide, of which 890 million were individuals living with obesity, and this number is expected to continue to rise.^4^ Obesity constitutes a major risk factor for various chronic diseases, including metabolic disorders such as type two diabetes, as well as cardiovascular complications like hypertension, dyslipidemia, and atherosclerosis.^5^ Moreover, obesity significantly impacts mental health and social well-being, increasing the risk of depression, anxiety, low self-esteem, and poor body image. It is also linked to social stigma, which can exacerbate mental health difficulties.^6,7^ Additionally, it can negatively impact well-being and quality of life, as well as reduce life expectancy.^8,9^

Despite advancements in pharmacological and surgical treatments, achieving and maintaining long-term weight loss remains challenging due to the complex and recurring nature of the condition.^3,5^ Studies have consistently highlighted the importance of healthy lifestyle behaviors, including a balanced diet, regular physical activity, effective stress management, and good sleep hygiene, in preventing and managing obesity and related chronic diseases.^10,11^ However, the effectiveness of these lifestyle factors is often influenced by individual, social, and economic circumstances, resulting in significant variability in outcomes.^12^ Recognizing the need for scalable, accessible, and cost-effective methods, mobile health (mHealth) technologies have emerged as promising tools for weight management.^13^ The World Health Organization defines mHealth interventions as “medical and public health practices supported by mobile devices, such as mobile phones, patient monitoring devices, personal digital assistance, and other wireless devices”.^14^ Taken together, this body of evidence underscores the growing interest in leveraging mHealth interventions to support sustainable lifestyle behavior change in the context of obesity management.

To optimize the efficacy of mHealth interventions, researchers emphasize the importance of leveraging theoretical frameworks that guide the development of digital health interventions and facilitate personalization of intervention components.^15,16^ Among these frameworks, Social Cognitive Theory (SCT) offers a particularly relevant lens for understanding behavior change processes within mHealth, as it conceptualizes behavior as the product of reciprocal interactions between personal, behavioral, and environmental factors.^17^ Within SCT, self-regulation and self-efficacy are central mechanisms underpinning sustained health behavior change. Self-regulation refers to individuals’ capacity to monitor, control, and adjust their thoughts, emotions, and behaviors in pursuit of health-related goals.^18^ This construct is especially salient in mHealth contexts, where interventions frequently incorporate Behavior Change Techniques (BCT) such as goal setting, self-monitoring, feedback, reminders, and social support to promote engagement and establish healthier routines.^19,20^ Evidence indicates that such mHealth components can enhance self-regulatory capacity by increasing self-awareness, supporting decision-making, and reinforcing motivation for lifestyle modification.^21^ Consistent with this, mHealth interventions integrating self-regulation techniques have demonstrated improvements in symptoms management, treatment adherence, and self-management across a range of chronic conditions, including obesity.^22,23^ Nevertheless, challenges related to user engagement and long-term adherence persist, underscoring the need for tailored intervention content and user-centered design approaches.^19,24^

Complementing the self-regulatory processes, self-efficacy represents a key motivational determinant that enables individuals to initiate and sustain behavior change. Defined as “the belief in one’s ability to plan and execute goal-directed actions”,^25^ self-efficacy has been associated with greater engagement in lifestyle modification, increased participation in health promoting behavior, and favorable health outcomes.^26,27^ Bandura emphasized the importance of integrating self-efficacy with self-regulation processes, as the dynamic interaction between them creates a powerful mechanism that can facilitate individuals’ successful achievement of their goals.^18^

While recent mHealth reviews on obesity and related conditions increasingly recognize the importance of self-regulation and self-efficacy, their primary focus remains on overall intervention effectiveness, technology features, or descriptive reporting of Behavior Change Techniques.^19,28,29^ Consequently, limited attention has been given to the systematic categorization of behavioral components, restricting the ability to link intervention content with specific behavioral mechanisms and to support theory-driven personalization.^30^ Moreover, existing evidence points to a lack of clear mapping between digital operationalization and specific Behavior Change Techniques, mechanisms of action, modes of delivery, and personalization strategies. This gap reduces design transparency and comparability across studies, which is essential for theoretical advancement, rigorous evaluation, and the scalable implementation of effective mHealth interventions.^30^

To address these gaps, this scoping review aims to identify and describe the components of mHealth interventions aimed at individuals living with obesity. Specifically, the review focuses on 1) identifying components related to self-regulation and self-efficacy, 2) mapping the operationalization of these components to modes of delivery, 3) exploring methods of personalization, and 4) identifying theoretical frameworks that inform intervention design. Furthermore, the review integrates a comprehensive mapping of digital operationalizations with a theory-informed categorization based on SCT. The review categorizes self-regulation components into five groups based on Bandura’s (1997) processes of self-regulation: self-monitoring, self-judgement, affective self-reaction, goal setting and planning, and self-regulation of motivation.^25^ Similarly, self-efficacy components are classified into four groups based on Bandura’s (1986) sources of self-efficacy: mastery experiences, vicarious experiences, social/verbal persuasion, and physiological/emotional experience.^17^ This approach offers a novel synthesis of how self-regulation and self-efficacy are operationalized in mHealth weight management interventions.

The research question is: What is the current state of knowledge regarding the mHealth intervention components that apply to self-regulation and self-efficacy in individuals with obesity? Given the broad nature of the research question and the primary objective of mapping available evidence on this topic, a scoping review was identified as the most appropriate methodological approach.

## Methods

The scoping review protocol was registered on the Open Science Framework (OSF; DOI: 10.17605/OSF.IO/HG6C9). The review was conducted in accordance with the Joanna Briggs Institute (JBI) methodology for scoping reviews^31^ and is reported following the PRISMA-ScR guidelines.^32^

### Search strategy and information sources

A systematic literature search was conducted in March 2025 across six databases: Scopus, PubMed, Web of Science, ProQuest, CINAHL, and PsychInfo. The search was performed by three researchers (ME, AA, SS) and employed predefined search strings, which were adapted to align with the specific functionalities and indexing of each database. The detailed search strategy is presented in the Supplemental Document 1.

All records retrieved from the databases were imported into the Rayyan AI platform,^33^ where automatic duplicate detection was applied. In the next stage, the same three reviewers (ME, AA, SS) independently screened titles and abstracts, while blinded to one another’s decisions. The papers were evenly distributed among reviewers and assessed against predefined inclusion and exclusion criteria (Table 1). To ensure consistent application of these criteria, a blinded rotating cross-examination procedure was used, whereby each reviewer independently re-screened a subset of studies initially assessed by another reviewer. Discrepancies were discussed collectively until consensus was reached. Full-text versions of potentially eligible studies were then retrieved and independently assessed by the same three reviewers using the same blinded rotating cross-examination procedure and predefined eligibility criteria. Any remaining disagreements were resolved through discussion.

**Table 1.** Predefined inclusion and exclusion criteria.

**Inclusion criteria**
• Studies focusing on adults aged ≥ 18 years living with obesity.
• Studies using mobile electronic devices (wearables, mobile apps, environmental sensors, etc.). Just-In-Time Adaptive Interventions (JITAI) and studies utilizing elements of Ecological Momentary Assessment (EMA) and Ecological Momentary Intervention (EMI).
• Focus on behavioral modification and self-regulation in obesity management.
• Diverse regions, contexts, and settings.
• Publications between 2015 and 2025.
• Full-length articles in English.
• Experimental and observational studies, including conference papers, study protocols, feasibility studies, proof-of-concept studies, and qualitative studies.
**Exclusion criteria**
• Preventative lifestyle interventions for general disease prevention unless they specifically target individuals with obesity.
• Studies on gestational weight management.
• Studies focusing on populations with severe health constraints (e.g. advanced chronic illnesses or severe physical/mental disabilities).

The search focused on studies published between 2015 and 2025 to encompass the development of contemporary mHealth interventions during a period characterized by substantial transformation in digital health. During this time, mHealth technologies evolved rapidly, driven by advances in mobile applications, wearable technologies, and digital health solutions.^34,35^

### Data extraction

To ensure alignment with the research question, the draft data-charting form was piloted on 20 studies identified through a preliminary literature search conducted in the ProQuest database. Pilot testing informed minor refinements to the form and accompanying guidance to improve the clarity and consistency of data charting.

The included studies were ordered by publication year in ascending order, from 2015 to 2025, and each study was assigned a study ID in the data charting form. Data extraction was conducted independently by four researchers (AD, RF, BK, OZ), with the included studies evenly allocated among them. An independent verification process was subsequently undertaken, in which the extracted data were evenly reallocated to three researchers (ME, AA, SS) for cross-examination to minimize errors and enhance reliability. Any discrepancies were discussed and resolved by consensus among ME, AA, and SS.

### Data synthesis

A narrative synthesis was used to summarize the current state of evidence, focusing on the principal concepts and characteristics of mHealth components related to self-efficacy and self-regulation. This approach facilitates an understanding of current trends, patterns, and potential gaps in existing research. Visual charts complement the narrative to enhance the clarity and interpretability of the findings.

## Results

### Selection of sources

**Figure 1.**
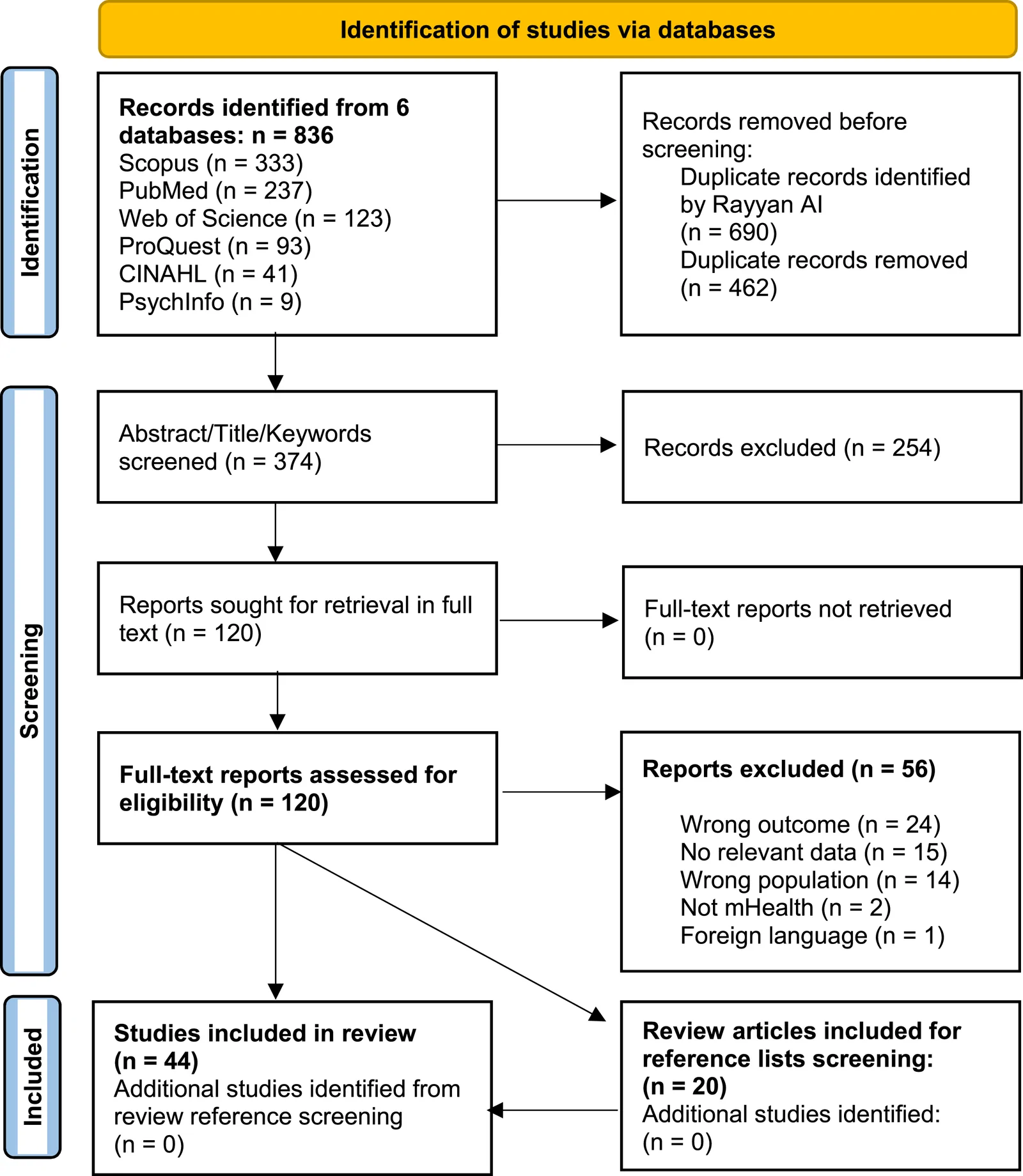
PRISMA flow diagram.

The initial database search yielded 836 articles. Rayyan AI identified 690 potential duplicate records, which were then reviewed and verified manually by the research team. Following this verification process, 462 duplicate records were confirmed as duplicates and removed prior to screening. Subsequently, 374 records were screened based on titles and abstracts. Of these, 254 were excluded, resulting in 120 potentially eligible studies. Full-text screening of these studies led to the exclusion of a further 56 articles, with reasons documented in the PRISMA flow diagram (Figure 1).

Of the remaining 64 articles, 44 met the eligibility criteria and were included in the review. The total of 20 articles were reviews and were therefore not eligible for inclusion. However, their reference lists were screened to identify potentially relevant studies. This process did not yield any additional eligible articles. Supplemental Document 2 presents the final data charting form consolidating findings across the included studies.

### Study characteristics

The included studies were predominantly conducted in North America, with the majority originating from the United States (59.1%), followed by papers from Europe (United Kingdom 6.8%; Germany 6.8%, Italy 2.3%, and Ireland 2.3%). Additional studies originated from Asia (China 4.5%, Singapore 4.5%, Japan 2.3%), Australia (6.8%), South America (Brazil 2.3%), and Oceania (New Zealand 2.3%).

Most studies (63.6%) were original studies reporting primary data collection, while the remaining studies involved secondary data analyses, study protocols, or conceptual papers. Study design varied, with randomized controlled trials being the most prevalent (56.8%). Although this distribution reflects a strong emphasis on experimental approaches, qualitative and mixed method designs also contributed to the evidence base (13.6%). Intervention durations ranged from 21 days to 24 months. Five studies (11.4%) focused specifically on young adults aged 18–35 years, while two studies (4.5%) targeted older adults aged 55 years and above. Gender-specific samples were uncommon, with one study (2.3%) focusing exclusively on women and three (6.8%) on men. Four studies (9%) targeted specific ethnic populations, including Hispanic, Southeast Asian, Caribbean, Central and South American, Māori, and Pacific communities. Additionally, ten studies (22.7%) addressed obesity within the broader context of various comorbidities, including metabolic (type 2 diabetes, hyperlipidemia), cardiovascular (hypertension), musculoskeletal (chronic pain), mental health (depression) and oncological conditions (cancer survivors).^36,37^

### Components related to self-regulation

Self-regulation components largely reflected Bandura’s (1997) self-regulatory framework.^25^ Table 2 provides an overview of mHealth components associated with self-regulation and their modes of delivery. Self-monitoring was the most consistently employed component, implemented via in-app food logs, food scanners, searchable food databases, as well as the syncing of objective data collected by mHealth technologies such as wearable devices and smartphones (e.g., Refs. 38 and 39). Reminders and notifications were frequently used to prompt users to self-monitor.^40^

**Table 2.** Mapping and categorization of mHealth components related to self-regulation and their operationalization across interventions.

Self-regulation processes	mHealth components related to self-regulation	Operationalization via mHealth technology
**Self-monitoring**	Monitoring behavior: diet, physical activity. Monitoring of outcomes: body weight, Body Mass Index.	In-app food logs and diaries, scanners and searchable food databases[1, 4, 10, 11, 14–16, 24, 25, 30, 39, 42] Automated calorie calculation [11] Wearable devices and smart scales for objective data sync: Fitbit, pedometers, heart rate trackers [6, 7, 12–14, 17, 21, 23, 25, 27–31, 34–36, 39–41, 43] Activity self-reports [16] Prompts and reminders to self-monitor [5]
**Goal setting and planning**	Intention formation. Goal setting: behavioral, outcome, personalized, adaptive. Action planning. Coping planning. Graded tasks. Goal revision/adaptation. Instructions on how to perform behavior. Context specific if-then planning, barrier management.	In-app goal setting tools [36] Automated personalized recommendations on realistic goals linked to self-monitoring features [11, 13, 16] Health coaching sessions (via videoconference, messaging, Zoom, in-app, online forums, etc.) for goal facilitation, revision, and management of barriers [17, 27, 29, 32, 35, 36] Skill mastering: through E-learning modules, videos, Smart Tips, Fact of the week, etc. [6, 14, 16, 36, 43]
**Self-judgement**	Discrepancy between current behavior and set goals. Provision of feedback on performance. Self-evaluation, self-reflection, and self-awareness.	Personalized feedback messages: automated or coach delivered [12, 14, 16, 17, 19, 22, 26–28, 34, 35, 39–41, 43, 44] Visualization through progress charts, weight graphs, and dashboards [6, 11–13, 15, 16, 26, 27, 29, 31, 32, 34, 36, 41] Algorithm driven feedback: color coded adherence flags and feedback [6] Warnings, e.g. caloric intake is too low, etc. [43] Chatbot-based check-ins on eating lapse triggers [42] Chatbot-based health information [38]
**Affective self-reaction**	Awareness of affective consequences: e.g. guilt, regret, etc. Affective state management/regulation. Stress management. Self-affirmation.	Mindfulness and cognitive defusion exercise (Acceptance and Commitment Therapy-based) [12] In-app reflection questions or cognitive prompts [36] Journal features to log feelings and situation-specific responses [4] Messaging/coaching on emotional understanding [5] Meal stopwatch [42]
**Self-regulation of motivation**	Reinforcement: social, contingent rewards, etc. Provision of general encouragement and verbal persuasion. Modeling opportunities and social support. Self-efficacy enhancement.	Gamification: point-based incentives, challenges, symbolic rewards, mastery badges [4, 5, 9, 13, 16, 19, 22, 24, 32, 37, 44] Social support features: online forums, peer message boards etc. [5, 8, 11, 16, 24–27, 31, 33–35, 37, 41, 44] Automated motivational messages and reminders [11, 12, 15] Choice architecture/nudge: default behavioral economics [22]

*Note.* Numbers shown in the table indicate the study ID assigned in the data charting form (Supplemental Document 2). Bibliographic details and the corresponding study IDs are provided in Table 5 in the Appendix.

Goal setting and planning were supported by automated, data driven tools, and personalized algorithms. An additional layer of personalization was provided through health coaching sessions via videoconferencing, messaging, and online forums, blending human interaction with digital delivery. Finally, both goal setting and planning were facilitated through education and instruction provision (e.g., with the help of e-learning modules, videos, smart tips and fact of the week messages).

Self-judgement was supported by the provision of feedback on performance and outcomes, fostering self-evaluation, self-reflection, and self-awareness. In most cases, it was heavily reliant on visualization through various graphical displays (progress charts, graphs, and dashboards), use of algorithm-driven feedback (e.g., color-coded adherence flags signaling compliance or non-compliance based on observed data). Furthermore, emerging conversational interfaces like chatbot-based check-ins on eating lapse triggers supported self-judgement by normalizing lapses, supporting self-reflection, and adaptive coping.^41^

Affective self-reaction was addressed through tools targeting emotional and stress regulation, including ACT-based (Acceptance and Commitment Therapy) mindfulness and cognitive defusion exercises to manage cravings and emotional eating.^38^ Reflective prompts, affective logging features, and diary functions further supported awareness of internal states. In some interventions, professional coaching addressed emotional understanding and coping (e.g., Refs. 42, 43 and 44). Simple tools, such as meal-timing features, promoted mindful eating and reduced emotionally driven consumption.^41^

Motivational self-regulation incorporated reinforcement, social support, and gamification strategies. Reinforcement in a form of social and contingent rewards to provide extrinsic motivation was often delivered through gamified challenges, mastery badges, and point-based incentives (e.g., Refs. 45 and 46). The rewards were often received for incremental changes and linked to self-monitoring activities. Social modelling and support were facilitated via peer forums, messaging boards, and virtual coaching (e.g., Refs. 47 and 48). One intervention used a concept from behavioral economics, specifically the choice architecture/nudges.^45^ Through this design, nudges provide cues that guide users towards their goals or healthier behavior without compromising their autonomy.^49^

### Components related to self-efficacy

Table 3 illustrates the application of components related to self-efficacy and their delivery modalities. All four sources of self-efficacy were represented. Mastery experiences were facilitated by graded tasks, opportunities for success and the recognition of achievements. These were delivered through visualization, automated feedback systems, updates on collected data, and challenges and competitions within apps or on social media (e.g., Refs. 50 and 51). Vicarious experiences were supported through social networks and online groups, where users can monitor and compare their progress against peers, engage with and read success stories, or access video demonstrations of desired behaviors (e.g., Refs. 52 and 53). Verbal persuasion was delivered via automated feedback, prompts, and encouragement through SMS, emails, or in-app notifications, as well as peer-to-peer exchanges (e.g., direct chats, online forums, and social media groups). Finally, physiological, and affective states were targeted through visualization, mindfulness practices, safety protocols, and coping tools such as stress management and distraction strategies (e.g., Refs. 54 and 55).

**Table 3.** Mapping and categorization of mHealth components related to self-efficacy and their operationalization across interventions.

Sources of self-efficacy	mHealth components related to self-efficacy	Ways of delivery via mHealth technology
**Mastery experiences**	Graded tasks. Providing opportunities for success. Acknowledging achievement. Instructions on how to perform behavior.	Visualization through progress charts, Smart Graphs, summaries and dashboards etc. [6, 11–13, 15, 16, 26, 27, 29, 31, 32, 34, 36, 41] Personalized feedback messages: automated or coach delivered [12, 14, 16, 17, 19, 22, 26–28, 34, 35, 39–41, 43, 44] Rewards for completing challenges: point-based incentives, symbolic rewards, mastery badges [4, 5, 9, 13, 16, 19, 22, 24, 32, 37, 44] Skill mastering: through E-learning modules, videos, Smart Tips, Fact of the week, etc. [6, 14, 16, 36, 43]
**Vicarious experiences**	Modelling behavior: observing others to succeed. Social comparison: opportunities to compare progress or behavior against peers. Demonstration: examples of how to perform a behavior.	Content: display of other users’ success stories [16, 44]. Comparing progress or behavior through online forums, groups’ message boards, shared online groups where peer activity are visible [5, 8, 11, 16, 24–27, 31, 33–35, 37, 41, 44] Videos: video demonstrations (e.g. exercises) via apps [16, 36, 43].
**Verbal persuasion**	Social support/Peer encouragement: receiving positive reinforcement or advice. Professional coaching. Personalized feedback.	Coaching/Consulting services: via videoconferencing or phone/Zoom [5, 17, 27, 29, 32, 35–37] Online podcasts and newsfeeds [5, 9, 11, 22] Messaging/Reminders/Prompts: Short Message Service, email, or in-app notifications providing encouragement, reinforcement, and prompts to action [5, 11, 12, 15, 20] Direct chats: online forums, social media groups, or direct online chats with peers or program professionals [5, 8, 11, 16, 24–27, 31, 33–35, 37, 41, 44]
**Physiological and affective states**	Affective state management/regulation. Managing risk/anxiety. Experiencing positive consequences through exerting desired behavior. Achievement acknowledgement. Visual imagery.	Mindfulness practice and cognitive diffusion exercises [12] Coping tools: In-app features e.g. distraction strategies, stress management [20, 22, 24] Automated safety instructions: clinical decision-tree algorithm delivered via digital voice assistant providing guidance based on self-reported data [43] Gamification: point-based incentives, challenges, etc. [4, 5, 9, 13, 16, 19, 22, 24, 32, 37, 44] Visual attractiveness [4, 20] Technological support [43] Virtual/Financial rewards/incentives [4, 5, 9, 13, 16, 19, 22, 24, 32, 37, 44]

*Note.* Numbers shown in the table indicate the study ID assigned in the data charting form (Supplemental Document 2). Bibliographic details and the corresponding study IDs are provided in Table 5 in the Appendix.

### Frequency of intervention components

**Figure 2.**
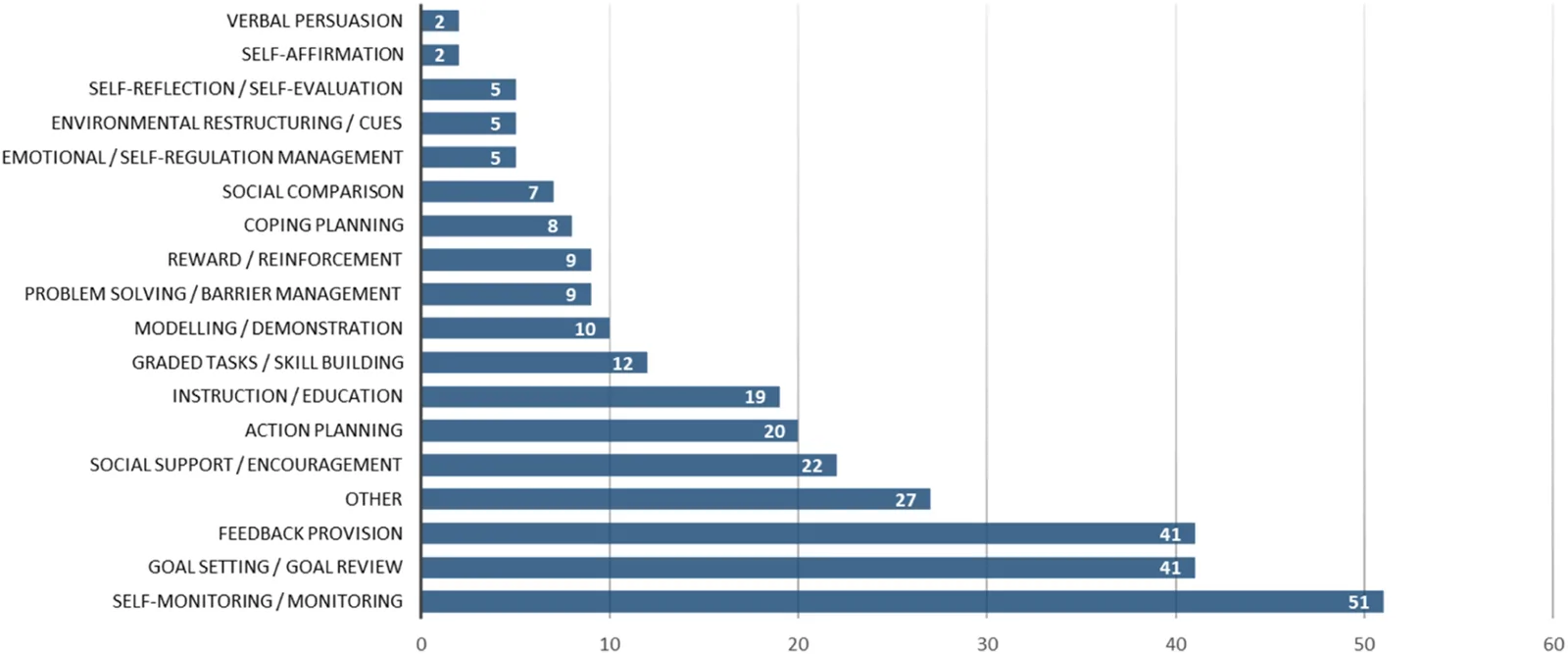
Frequency of intervention components related to self-regulation and self-efficacy.

Figure 2 presents a summary of the frequency of intervention components related to self-regulation and self-efficacy across the included studies. Monitoring and self-monitoring were the most frequently implemented intervention components (n = 51), followed by goal setting/goal review (n = 41) and feedback provision (n = 41). The “Other” category (n = 27) comprises intervention components that were each reported only once across included studies, such as behavioral contract, the use of credible source, mental imaginary, safety-related components, just to name a few.

### Personalization

Personalization occurred across behavioral, contextual, and content dimensions. Baseline assessments and self-monitoring data informed personalized goal setting and feedback (e.g., Refs. 56 and 57). Wearable sensors enabled continuous adaptation of goals and intervention content based on users’ progress and needs, rather than as fixed targets (e.g., Refs. 58 and 59). Data from wearables and self-monitoring also inform the delivery of personalized feedback. Feedback was tailored to highlight strengths, address challenges, and celebrate milestones, typically delivered through visual progress charts and algorithm-driven suggestions tailored to the individual (e.g., Refs. 53 and 60).

Reminders, prompts, and nudges were tailored to users’ immediate context and recent behaviors, drawing on ecological momentary intervention principles to optimize timing and relevance (e.g., Refs. 42 and 61). Content delivery was tailored to the participant´s stage of change and perceived barriers. Participants received articles, videos, or practical tips that addressed specific gaps in knowledge or skills (e.g., Refs. 52 and 62). Social personalization included peer matching, relevant social comparison, and tailored incentives (e.g., Refs. 50 and 57). While algorithms automated many personalization processes, human interaction complemented these systems (e.g., Refs. 58 and 63). For some populations, personalization also incorporated socio-cultural context (e.g., Refs. 46 and 64).

### Theoretical frameworks

Slightly over half of the included studies (25/44; 56.8%) explicitly grounded their interventions in established behavioral theories. SCT was the most frequently applied framework (15/25; 60%), followed by the Transtheoretical Model (4/25; 16%). In seven studies (7/25; 28%), theoretical frameworks were combined to address behavior change across individual and contextual levels, including integrations of SCT with Kanfer’s self-regulation theory or Self-Determination Theory.

### Types of mHealth technologies

Between 2015 and 2025, mHealth technologies evolved from basic tracking and communication tools to more integrated, AI-enhanced systems. Earlier studies (2015 to 2020) utilized primarily mobile applications, wearable devices, and simple communications systems. Applications such as B-Mobile, My Fitness Pal, Livestrong, Spark People, and DietMate Pro© were used for self-reporting, tracking and content delivery.^39,61,62^ Wearable devices including pedometers, accelerometers (e.g., Refs. 65 and 66), and Fitbit trackers/watches (e.g., Refs. 59 and 67) were used for objective monitoring of physical activity. Simple communication systems such as SMS, interactive voice response calls, and in-app messaging provided reminders and support.^63^ Ecological Momentary Intervention (EMI) was reported once in 2016 in combination with a smartphone app, smart scale, and wearable device.^56^

From 2021 onwards, many mHealth technologies have evolved toward more integrated, AI-enhanced, and sensor-based systems. Applications such as KENPO and eTrip incorporated AI-driven chatbots, image recognition, and automated reminders.^41,68^ Applications like Zanadio and FoodSmart functioned as integrative platforms connecting wearables, Bluetooth scales, and online food services.^45,57^ Voice-activated technologies emerged, exemplified by the integration of Amazon Alexa with software portals.^55,65^ New devices tracked additional health metrics, such as the Mio-Fuse heartbeat tracker for cardiovascular monitoring and glucometers for behavioral feedback.^45,65^ Data aggregation platforms like Fitabase enabled integration of data from wearables and smart scales to trigger personalized messaging.^66^ Social and communication platforms, including Facebook, Messenger, and WeChat, were used for feedback and social support.^44,50^

Unlike other mHealth technologies, smart scales, both generic and branded (e.g., Body Trace, Tanita, Fitbit Aria)^69–71^, were used for weight tracking across the entire study period 2015-2025 (e.g., Refs. 72–74).

### Hybrid modes of delivery

Over half of the included interventions (54.4%) combined mHealth technologies with additional delivery modes, resulting in hybrid intervention approaches. Structured in-person group sessions were employed to provide educational content and social support (e.g., Refs. 63 and 75). Human interaction was also facilitated through remote check-in calls, group calls, and chat-based discussions, ensuring continuous motivational reinforcement through personalized feedback and individualized problem solving (e.g., Refs. 76 and 77). In some cases, printed materials, including handbooks, reports, and diaries/logs, were utilized to facilitate education and record behaviors offline (e.g., Refs. 64 and 72).

## Discussion

The scoping review aimed to explore the existing literature on mHealth interventions targeting adults living with obesity, with four primary objectives: (1) to identify intervention components related to self-regulation and self-efficacy; (2) to examine how these components are operationalized through mHealth technologies; (3) to explore personalization approaches; and (4) to identify the theoretical frameworks underpinning intervention design. Given its broad mapping focus, the review sought to synthesize and organize available evidence rather than evaluate study quality, with the overarching goal of informing future research and the development of more theory-informed, personalized mHealth interventions for obesity management.

### Self-regulation

The findings of the scoping review indicate that components related to self-regulation are widely incorporated into mHealth interventions designed to support engagement in healthy lifestyle routines.^19,20^ Previous research suggest that such mHealth components may support self-regulatory capacity by increasing self-awareness, supporting decision-making, and reinforcing motivation.^21^ Nevertheless, challenges related to sustained engagement and long-term adherence persist. This underscores the importance of tailoring intervention content to individual needs and integrating user-centered design principles.^19,24^ Understanding how mHealth tools operationalize self-regulation processes is therefore essential for designing effective, scalable, and personalized interventions for individuals living with obesity.

### Self-efficacy

All four sources of self-efficacy as defined by Bandura (1986) were represented across the examined mHealth interventions.^17^ Graded tasks, progress tracking, visualization, automatic feedback, and digital prompts were among the components related to mastery experiences identified across the included studies. Previous studies have suggested that these approaches may facilitate mastery experiences in mHealth interventions. For example, a meta-analysis of RCTs examining studies that utilized mobile applications for weight management among individuals with overweight or obesity identified self-monitoring, provision of instructions, feedback, goal setting, and action planning as the most frequently employed BCTs. In addition, the use of mobile phone applications was associated with a significant reduction in body weight in the same meta-analysis.^73^ These techniques align with established psychological mechanisms related to self-efficacy by supporting individuals’ perception of behavioral control, providing structured guidance for action, and enabling systematic monitoring of progress.

Vicarious experiences, characterized by learning and gaining motivation through the observation of others, represent an important source of self-efficacy within social-cognitive theory.^17^ Although direct research on the role of vicarious experiences in mHealth interventions and among individuals with obesity is limited, some interventions employ components such as progress sharing, group challenges, or community forums.^74,76^ These may enable participants to engage with social networks, forums, messages, or instructional videos, facilitating the observation of others achieving change. Results show that verbal persuasion was implemented through personalized messages, notifications, coaching, or peer communication (e.g., Refs. 63 and 78). These findings align with a meta-analysis of mobile weight loss applications, which identified self-monitoring, instructional guidance, and feedback are among the most frequently employed BCTs.^73^ Future studies should explicitly operationalize and experimentally test modelling-facilitative components to clarify their role in activating social learning processes and enhancing self-efficacy in digital behavior change interventions.

Our review indicates that mHealth tools frequently incorporate features such as visualization, mindfulness practices, coping strategies, gamification elements, or safety-related algorithms. These components can be mapped onto physiological and affective states as sources of self-efficacy and have been described in previous literature as supporting anxiety reduction, emotional regulation, and perception of control.^79–81^ While data visualization enhances user engagement, it is crucial to ensure that end users accurately comprehend the presented data. A study by Alshehhi et al. (2023) found that participants prefer bar and pie charts combined with text summaries for verification.^78^ However, the rapid emergence of AI-based approaches may substantially alter this landscape, underscoring the need to examine how adaptive, data-driven systems influence users’ interpretation of feedback and regulation of physiological and affective state. In the context of mental health, mindfulness techniques have been shown to reduce stress and improve quality of life, which has been evidenced in various Randomized Controlled Trials.^82,83^ In addition, stress management strategies that are often embedded within cognitive behavioral therapy frameworks represent an additional evidence-based approach. A literature review of 34 studies identified specific mHealth features, such as thought recording, stress management cards, and self-monitoring tools, as mechanisms supporting stress management and mental health outcomes.^84^ Gamification is commonly employed as a component related to self-efficacy, user engagement and adherence. Previous literature has associated gamification features with positive affective states, particularly user satisfaction. Common features include digital rewards, challenges, leaderboards, and motivational messages.^85^

### Personalization

Although personalization appeared frequently across interventions, most implementations relied on static tailoring rather than genuinely adaptive strategies.^86^ In many cases, goals or recommendations were tailored only from demographic or baseline data (e.g., Refs. 64 and 87), whereas comparatively few interventions used iterative assessments or user-modifiable plans (e.g., Refs. 59 and 68). As a result, personalization tended to function as a design feature rather than an evolving, mechanism-driven component of behavior change.

Despite widespread use of wearable devices, sensor data were used for passive monitoring rather than active adaptation. Only a small number of interventions adjusted goals or feedback based on real-time metrics such as step counts, activity distribution, heart-rate reserve, or blood glucose levels, and even these adaptations followed simple rule-based logic.^52,67^ This suggests that sensor-driven personalization remains an underutilized capability within mHealth systems. The integration of clinical parameters was similarly limited: aside from one glucose-responsive decision-tree and isolated examples of electronic health record-informed goal setting,^63,65^ most interventions did not incorporate clinical profiles into their tailoring logic, reducing their relevance for multimorbid populations.

Personalized reminders and timing adjustments were present but generally user-defined rather than system-driven, and no study optimized message timing based on behavioral patterns or predicted risk windows. Consequently, informational tailoring remained preference-based rather than dynamically optimized. Future advances in AI may enable personalization to function as a true mechanism of behavior change, with adaptive systems continuously updating goals, feedback, and timing based on real-time behavioral, physiological, and contextual data.

Terminology was also inconsistent. Some interventions described baseline tailoring as personalized, whereas others used far more sophisticated adaptive features but reported them only briefly. Although baseline tailoring is a valid form of personalization, the lack of distinctions between levels of personalization complicates synthesis and highlights the need for clearer reporting standards and structured taxonomies.

### Theoretical frameworks

Slightly more than half of the interventions were grounded in established behavioral theories, while a substantial proportion lacked explicit theoretical foundations. This limits interpretability and the ability to identify mechanisms linking intervention components to outcomes, reflecting long-standing concerns about superficial or inconsistent theoretical application.^87^ Several interventions relied solely on behavior change techniques without specifying an overarching model, reinforcing the distinction between techniques and theory. Among theory-based interventions, Social Cognitive Theory predominated, followed by Transtheoretical Model, reflecting limited theoretical diversity and a concentration around a narrow conceptual approach.

Although some studies combined multiple theories, integration was often insufficiently articulated, and theoretical constructs were rarely mapped to specific intervention components, increasing the risk of conceptual ambiguity. Moreover, many commonly used behavior change theories are not specified at a level that readily supports the implementation and modeling of dynamic, time-varying processes, limiting their applicability in digital contexts. In the mHealth context, this is particularly problematic, as digital behavior change often requires frameworks capable of addressing dynamic tailoring, engagement, and real-time contextual influences, such as COM-B/Behavior Change Wheel or Persuasive Systems Design.^88–91^ Limited use of such frameworks further constraints the potential of mHealth systems to support adaptive intervention delivery and continuous monitoring.

### Types of technologies in mHealth

The review findings reveal heterogeneity in the types of technologies used within mHealth interventions targeting adults living with obesity, alongside a clear temporal progression in technological sophistication. Similar heterogeneity has been reported in previous review of digital weight management interventions, which describe a wide range of technological approaches differing in complexity, functionality, and level of integration.^92^

Early interventions predominantly employed basic digital tracking and communication tools, enabling self-monitoring and the provision of basic prompts or feedback. This focus on self-monitoring aligns with earlier evidence identifying self-monitoring as a foundational behavior change strategy in mHealth-supported weight management. Burke et al. (2012) previously stated that self-monitoring of dietary intake, physical activity, and weight is a key strategy in behavioral weight loss interventions and digital methods have shown potential for increasing adherence and behavior change.^93^

More recent interventions increasingly incorporated integrated, sensor-enhanced, and AI-supported systems, reflecting broader technological developments in digital health. Prior study from Smits et al. (2025) have emphasized that the integration of wearable sensors, automated data processing, and intelligent feedback systems enables more responsive and contextually relevant intervention delivery through automated feedback loops, improved scalability, and reduced reliance on manual input.^94^

### Hybrid delivery: Combination of technology and human support

Consistent with prior evidence, hybrid interventions combining mHealth technologies with human support appeared particularly promising. It allows for both scalability and depth of response to individual challenges, such as overcoming motivational dips or lapse loops. A systematic review from Hamine et al. (2015) shows that interventions combining technological components with human contact tend to achieve better adherence and outcomes compared with purely digital.^13^ Future research should therefore prioritize the systematic evaluation of hybrid delivery models, including the identification of optimal combinations and intensities of technological and human components. In parallel, continued progress in adaptive algorithms and sensor integration offers an opportunity to advance dynamic personalization and move beyond static tailoring within mHealth interventions.

### Reporting standards

A key finding of this review was substantial heterogeneity in reporting mHealth components, particularly behavioral science elements. Babatunde et al. (2025) highlighted inconsistencies in reporting, noting that the quality, completeness, and objectivity of the current evidence base for mHealth interventions vary considerably, making it challenging to effectively compare intervention strategies.^95^ Consistent with these observations, the findings of this scoping review emphasize the need for the standardized reporting of mHealth interventions to enhance the quality of future research.

While the reviewed mHealth interventions demonstrated an implicit use of theoretical concepts of self-efficacy and self-regulation, the review team frequently had to identify these features through functional descriptions rather than explicit theoretical labelling. This failure to explicitly name or define these elements confirms the urgent need for standardized reporting guidelines, as it can compromise replicability, limit the validity of future meta-analyses, and limit the ability to accurately interpret mechanisms of action. Although mHealth interventions are progressively relying on behavioral science principles, the reporting of theoretical foundations, behavioral constructs, and their mechanisms of action remain inconsistent. The scoping review demonstrates that this is not an isolated exception, but it represents a recurring structural weakness in current mHealth reporting practice.

The mERA (Mobile Health Evidence Reporting and Assessment) guidelines require authors of mHealth interventions to describe the content, context, and technical aspects of their interventions. This is achieved through 16 core items, which encompass aspects such as infrastructure, platform, interoperability, delivery, feasibility, scalability, data security, among others.^96^ This is particularly valuable because generic trial guidelines, such as SPIRIT 2025 (Standard Protocol Items: Recommendations for Interventional Trials) and CONSORT (Consolidated Standards of Reporting Trials), or observational study guidelines, such as STROBE (Strengthening the Reporting of Observational Studies in Epidemiology), do not address infrastructural and technical factors.^97–99^ Similarly, the CREMAS guidelines (Checklist for Reporting Ecological Momentary Assessment Studies) complement design-specific tools and are important attempts to close the gap for EMA and geographically explicit EMA.^100^ However, these extended guidelines do not offer support to authors in explaining why and how intervention is expected to influence behavior.

Based on our observations, we formulated a set of core reporting recommendations that directly address the most frequent gaps encountered during the review. These recommendations focus on four fundamental elements: 1) explicit identification of theoretical framework or construct driven rationale guiding the intervention, 2) clear specification of the taxonomic and conceptual system used to standardize constructs and techniques, 3) structured mapping of intervention components to behavioral constructs and corresponding behavior change techniques, and 4) explicit description of the hypothesized operationalization linking intervention features to behavior change. Together, these elements represent the minimal conceptual information that was consistently missing across the reviewed studies, yet they are essential for understanding how and why mHealth intervention is expected to change behavior. To support authors in implementing these principles, Table 4 organizes recommendations into a practical format for effective reporting. This table is intended as a supportive tool for researchers who integrate behavioral science elements into mHealth interventions targeting behavior change.

**Table 4.** Reporting recommendations for underreported behavioral science elements in mHealth interventions.

Underreported elements	Reporting recommendations	Added value for reporting
Theoretical Framework or Construct	Authors should name the behavioral theory or framework that informs the intervention. If no full theory is used, clarify whether the design is construct-driven and if so, define each behavioral construct used to guide intervention design.	This may help to enhance reproducibility and contextualization and therefore improve the quality of evidence.
Concept	Authors should identify the taxonomy or conceptual systems, such as BCTTv1 for Behavior Change Techniques, the TDF or Mechanisms of Action taxonomy for constructs.	This may enable scientific communication, synthesis and analysis of finding across studies, improvement of intervention fidelity and replicability, and it can address multidisciplinary complexity.
Intervention component – Construct – BCT Mapping	Authors should present a structured table linking each intervention component or digital feature (e.g. prompts, notifications etc.) to its corresponding behavioral construct and BCT code (e.g. BCTTv1).	This may facilitate verification of the theoretical logic, replicability, and synthesis and analysis of findings across studies.
Mechanism logic	Authors should describe the hypothesized mechanisms of action linking intervention features to behavior change.	This may promote robust research design and enhance scalability and adaptability to different populations, health needs, or geographical context.

*Notes.* BCT: Behavior Change Technique; BCTTv1: Behavior Change Technique Taxonomy version 1; TDF: Theoretical Domains Framework.

### Strengths and limitations

The temporal restriction of the review to studies published from 2015 to 2025 strengthens the relevance of the evidence base, reflecting the rapidly evolving nature of the mHealth field. Furthermore, the review contrasts earlier studies from 2015-2020 with those published between 2021-2025, thereby capturing emerging trends, technological advancements, and the development of core mHealth components over time. Furthermore, through comprehensive mapping, the scoping review offers key insights into the various approaches to supporting self-regulation and self-efficacy through mHealth interventions and therefore, it offers a theoretical and practical foundation for designing mHealth interventions. A limitation of this review is that only studies published in English were included, which may have resulted in the exclusion of potentially relevant studies and increased the risk of language bias. A key challenge in this scoping review stems from the inconsistent and often insufficient reporting of mHealth components in the included studies, particularly with respect to behavioral elements. Although included studies appeared to draw on constructs such as self-regulation and self-efficacy, these were rarely reported explicitly. Consequently, the review team was required to identify theoretical components based on functional description, which may have affected the precision of the synthesis of mechanisms of action. In response to this limitation, a set of reporting recommendations designed to support authors in strengthening the transparency and consistency of behavioral science reporting in mHealth research was proposed.

## Conclusion

The scoping review highlights the evolving nature of mHealth interventions targeting adults living with obesity, emphasizing the critical role of self-regulation and self-efficacy in weight management. This review systematically categorizes intervention components related to self-regulation and self-efficacy according to Banduras’ framework and maps their operationalization to specific digital delivery features. This approach provides a novel, structured evidence base that enables researchers to more precisely align intervention content with specific behavioral mechanisms and user needs, therefore strengthening theory driven personalization in mHealth interventions. Continued research should focus on enhancing dynamic personalization, expanding theoretical diversity, and rigorously evaluating hybrid models of delivery. Notably, inconsistent reporting of behavioral elements remains a limitation, which this review addresses by proposing recommendations to improve transparency and standardization in future mHealth studies.

## Supplemental material


Supplemental Material - Use of mHealth interventions to enhance self-regulation and self-efficacy in individuals living with obesity: Scoping review
Supplemental Material for Use of mHealth interventions to enhance self-regulation and self-efficacy in individuals living with obesity: Scoping review by Marcela Ely, Asghar Ahmadi, Syed M. Shariq, Andrea Duchonova, Roman Filo, Barbora Kastovska, Ondrej Zach, Holly B. Jimison and Steriani Elavsky in Digital Health.

Supplemental Material - Use of mHealth interventions to enhance self-regulation and self-efficacy in individuals living with obesity: Scoping review
Supplemental Material for Use of mHealth interventions to enhance self-regulation and self-efficacy in individuals living with obesity: Scoping review by Marcela Ely, Asghar Ahmadi, Syed M. Shariq, Andrea Duchonova, Roman Filo, Barbora Kastovska, Ondrej Zach, Holly B. Jimison and Steriani Elavsky in Digital Health.

Supplemental Material - Use of mHealth interventions to enhance self-regulation and self-efficacy in individuals living with obesity: Scoping review
Supplemental Material for Use of mHealth interventions to enhance self-regulation and self-efficacy in individuals living with obesity: Scoping review by Marcela Ely, Asghar Ahmadi, Syed M. Shariq, Andrea Duchonova, Roman Filo, Barbora Kastovska, Ondrej Zach, Holly B. Jimison and Steriani Elavsky in Digital Health.

## Data Availability

Data charting form provided as Supplementary Document 2.*

## Appendix

**Table 5.** Study ID and bibliographic overview of included studies.

ID	Year	Authors	Title of source
1	2015	Ambeba EJ, Ye L, Sereika SM, Styn MA, Acharya SD, Sevick MA, Ewing LJ, Conroy MB, Glanz K, Zheng Y, Goode RW, Mattos M, Burke LE	The use of mHealth to deliver tailored messages reduces reported energy and fat intake.
2	2015	Lin PH, Intille S, Bennett G, Bosworth HB, Corsino L, Voils C, Grambow S, Lazenka T, Batch BC, Tyson C, Svetkey LP	Adaptive intervention design in mobile health: Intervention design and development in the Cell Phone Intervention for You trial.
3	2015	Thomas, Graham, J., Bond, D.S.	Behavioral response to a just-in-time adaptive intervention (JITAI) to reduce sedentary behavior in obese adults: Implications for JITAI optimization
4	2015	Tang J, Abraham C, Stamp E, Greaves C	How can weight-loss app designers’ best engage and support users? A qualitative investigation.
5	2015	Hales, Sarah B.	Refinement and pilot testing social networks for encouraging healthy behaviors: The social pounds off digitally (social POD) study
6	2016	Martin CK, Gilmore LA, Apolzan JW, Myers CA, Thomas DM, Redman LM	Smartloss: A Personalized Mobile Health Intervention for Weight Management and Health Promotion.
7	2016	Foley P, Steinberg D, Levine E, Askew S, Batch BC, Puleo EM, Svetkey LP, Bosworth HB, DeVries A, Miranda H, Bennett GG	Track: A randomized controlled trial of a digital health obesity treatment intervention for medically vulnerable primary care patients.
8	2016	Hales, Sarah, Turner-McGrievy, Gabrielle, Fahim, Arjang, Freix, Andrew, Wilcox, Sara, Davis, Rachel E, Huhns, Michael, Valafar, Homayoun	A Mixed-Methods Approach to the Development, Refinement, and Pilot Testing of Social Networks for Improving Healthy Behaviors
9	2017	Hales S, Turner-McGrievy GM, Wilcox S, Davis RE, Fahim A, Huhns M, Valafar H	Trading pounds for points: Engagement and weight loss in a mobile health intervention.
10	2017	Burke LE, Zheng Y, Ma Q, Mancino J, Loar I, Music E, Styn M, Ewing L, French B, Sieworek D, Smailagic A, Sereika SM	The SMARTER pilot study: Testing feasibility of real-time feedback for dietary self-monitoring.
11	2017	Kim H, Faw M, Michaelides A	Mobile But Connected: Harnessing the Power of Self-Efficacy and Group Support for Weight Loss Success through mHealth Intervention.
12	2018	Cattivelli R, Castelnuovo G, Musetti A, Varallo G, Spatola CAM, Riboni FV, Usubini AG, Tosolin F, Manzoni GM, Capodaglio P, Rossi A, Pietrabissa G, Molinari E	ACTonHEALTH study protocol: promoting psychological flexibility with activity tracker and mHealth tools to foster healthful lifestyle for obesity and other chronic health conditions.
13	2018	Korinek, Elizabeth V, Phatak, Sayali S, Martin, Cesar A, Freigoun, Mohammad T, Rivera, Daniel E, Adams, Marc A, Klasnja, Pedja, Buman, Matthew P, Hekler, Eric B	Adaptive step goals and rewards: a longitudinal growth model of daily steps for a smartphone-based walking intervention
14	2018	Duncan MJ, Brown WJ, Burrows TL, Collins CE, Fenton S, Glozier N, Kolt GS, Morgan PJ, Hensley M, Holliday EG, Murawski B, Plotnikoff RC, Rayward AT, Stamatakis E, Vandelanotte C	Examining the efficacy of a multicomponent m-Health physical activity, diet and sleep intervention for weight loss in overweight and obese adults: randomised controlled trial protocol.
15	2018	Young, Myles D, Morgan, Philip J	Effect of a Gender-Tailored eHealth Weight Loss Program on the Depressive Symptoms of Overweight and Obese Men: Pre-Post Study
16	2018	Beleigoli AM, Queiroz de Andrade A, Haueisen Diniz MF, Alvares RS, Ribeiro AL	Online platform for healthy weight loss in adults with overweight and obesity - the “POEmaS” project: a randomized controlled trial.
17	2019	Johnson, K.E., Alencar, M.K., Coakley, K.E., Swift, D.L., Cole, N.H., Mermier, C.M., Kravitz, L., Amorim, F.T., Gibson, A.L.	Telemedicine-Based Health Coaching Is Effective for Inducing Weight Loss and Improving Metabolic Markers
18	2019	Batsis JA, Zagaria AB, Halter RJ, Boateng GG, Proctor P, Bartels SJ, Kotz D	Use of Amulet in behavioral change for geriatric obesity management.
19	2019	Corrigan, Callie, Dalhousie, Sally, Firestone, Ridvan, Funaki, Tevita, Goodwin, Debbie, Grey, Jacqui, Akarere Henry, Humphrey, Gayl, Jull, Andrew, Vano, Mereaumate, Pekepo, Crystal, Lisa Te Morenga, Whittaker, Robyn, Cliona Ni Mhurchu	Using codesign to develop a culturally tailored, behavior change mHealth intervention for indigenous and other priority communities: A case study in New Zealand
20	2019	Kliemann N, Croker H, Johnson F, Beeken RJ	Development of the Top Tips Habit-Based Weight Loss App and Preliminary Indications of Its Usage, Effectiveness, and Acceptability: Mixed-Methods Pilot Study.
21	2020	Valle, C.G., Nezami, B.T., Tate, D.F.	Designing in-app messages to nudge behavior change: Lessons learned from a weight management app for young adults
22	2020	Hu EA, Nguyen V, Langheier J, Shurney D	Weight Reduction Through a Digital Nutrition and Food Purchasing Platform Among Users With Obesity: Longitudinal Study.
23	2020	Burke LE, Sereika SM, Parmanto B, Beatrice B, Cajita M, Loar I, Pulantara IW, Wang Y, Kariuki J, Yu Y, Cedillo M, Cheng J, Conroy MB	The SMARTER Trial: Design of a trial testing tailored mHealth feedback to impact self-monitoring of diet, physical activity, and weight.
24	2020	Buchan K, Morgan HM	Using the Onitor (®) Track for weight loss: A mixed methods study among overweight and obese women.
25	2021	Carey A, Yang Q, DeLuca L, Toro-Ramos T, Kim Y, Michaelides A	The Relationship Between Weight Loss Outcomes and Engagement in a Mobile Behavioral Change Intervention: Retrospective Analysis.
26	2021	Gans KM, Dulin A, Palomo V, Benitez T, Dunsiger S, Dionne L, Champion G, Edgar R, Marcus B	A Tailored Web- and Text-Based Intervention to Increase Physical Activity for Latino Men: Protocol for a Randomized Controlled Feasibility Trial.
27	2021	Fanning, Jason, Brooks, Amber K, Hsieh, Katherine L, Kershner, Kyle, Furlipa, Joy, Nicklas, Barbara J, Rejeski, W Jack	Building on Lessons Learned in a Mobile Intervention to Reduce Pain and Improve Health (MORPH): Protocol for the MORPH-II Trial.
28	2021	Walsh, Jane C, Richmond, Janice, Jenny Mc Sharry, Groarke, AnnMarie, Glynn, Liam, Kelly, Mary Grace, Harney, Owen, Groarke, Jenny M	Examining the Impact of an mHealth Behavior Change Intervention With a Brief In-Person Component for Cancer Survivors With Overweight or Obesity: Randomized Controlled Trial.
29	2022	Garcia, D.O., Valdez, L.A., Aceves, B., Bell, M.L., Rabe, B.A., Villavicencio, E.A., Marrero, D.G., Melton, F., Hooker, S.P.	mHealth-Supported Gender- and Culturally Sensitive Weight Loss Intervention for Hispanic Men With Overweight and Obesity: Single-Arm Pilot Study.
30	2022	Burke, L.E., Sereika, S.M., Bizhanova, Z., Parmanto, B., Kariuki, J., Cheng, J., Beatrice, B., Cedillo, M., Pulantara, I.W., Wang, Y., Loar, I., Conroy, M.B.	The Effect of Tailored, Daily, Smartphone Feedback to Lifestyle Self-Monitoring on Weight Loss at 12 Months: the SMARTER Randomized Clinical Trial
31	2022	Brannon GE, Ray M, Cho P, Baum M, Beg MS, Bevers T, Schembre SM, Basen-Engquist K, Liao Y	A qualitative study to explore the acceptability and usefulness of personalized biofeedback to motivate physical activity in cancer survivors.
32	2022	Alexander Wilhelm Gorny, Wei Chian Douglas Chee, Müller-Riemenschneider, Falk	Active Use and Engagement in an mHealth Initiative Among Young Men With Obesity: Mixed Methods Study
33	2022	Hu Y, Zhang Y, Qi X, Xu X, Rahmani J, Bai R, Mei Y	Supervised mHeath Exercise Improves Health Factors More Than Self-Directed mHealth Exercise: A Clinical Controlled Study
34	2022	Drew RJ, Morgan PJ, Young MD	Mechanisms of an eHealth program targeting depression in men with overweight or obesity: A randomised trial.
35	2022	Mansour-Assi SJ, Golaszewski NM, Costello VL, Wing D, Persinger H, Coleman A, Lytle L, Larsen BA, Jain S, Weibel N, Rock CL, Patrick K, Hekler E, Godino JG	Social Mobile Approaches to Reducing Weight (SMART) 2.0: protocol of a randomized controlled trial among young adults in university settings.
36	2022	Forkmann, K., Roth, L., Mehl, N.	Introducing zanadio—A Digitalized, Multimodal Program to Treat Obesity
37	2023	Lin H, Xie S, Xu D, Wu F, Huang R, Wu H, Zhang Y, An J, Yang M, Deng N	Evaluation of a workplace weight management program based on WeChat platform for obese/overweight people in China using the RE-AIM framework.
38	2023	Sakane, Naoki, Suganuma, Akiko, Domichi, Masayuki, Shin Sukino, Abe, Keiko, Fujisaki, Akiyoshi, Kanazawa, Ai, Sugimoto, Mamiko	The Effect of a mHealth App (KENPO-app) for Specific Health Guidance on Weight Changes in Adults With Obesity and Hypertension: Pilot Randomized Controlled Trial.
39	2023	Cheng J, Costacou T, Sereika SM, Conroy MB, Parmanto B, Rockette-Wagner B, Kriska AM, Klem ML, Burke LE	Effect of an mHealth weight loss intervention on Healthy Eating Index diet quality: the SMARTER randomised controlled trial.
40	2024	Jacob K Kariuki, Zhadyra Bizhanova, Molly B Conroy, Lora E Burke, Jessica Cheng, Britney Beatrice, Susan M Sereika	The Association between Neighborhood Walkability and Physical Activity in a Behavioral Weight Loss Trial Testing the Addition of Remotely Delivered Feedback Messages to Self-Monitoring
41	2024	Gerber S, Silver RE, Das SK, Greene SS, Dix SR, Ramirez I, Morcos CL, Dao MC, Ceglia L, Roberts SB	Development and Feasibility of an eHealth Diabetes Prevention Program Adapted for Older Adults-Results from a Randomized Control Pilot Study.
42	2024	Chew HSJ, Chew NW, Loong SSE, Lim SL, Tam WSW, Chin YH, Chao AM, Dimitriadis GK, Gao Y, So JBY, Shabbir A, Ngiam KY	Effectiveness of an Artificial Intelligence-Assisted App for Improving Eating Behaviors: Mixed Methods Evaluation.
43	2024	Glavas, Costas, Scott, David, Sood, Surbhi, George, Elena S, Daly, Robin M, Gvozdenko, Eugene, de Courten, Barbora, Jansons, Paul	Exploring the Feasibility of Digital Voice Assistants for Delivery of a Home-Based Exercise Intervention in Older Adults With Obesity and Type 2 Diabetes Mellitus: Randomized Controlled Trial
44	2025	Haderer M, Hofmann R, Bartelmeß T, König L, Betz C, Al Masri M, Bader A, von Schau N	General practitioner-centered rural obesity management: Design, protocol and baseline data of the German HAPpEN pragmatic trial.
