# Supplementary material for: Use of mHealth interventions to enhance self-regulation and self-efficacy in individuals living with obesity: Scoping review
Source: Digit Health. 2026 Aug 19;12:20552076261474266. doi: 10.1177/20552076261474266 (PMC13490795; doi:10.1177/20552076261474266)
Supplement: Supplementary file 1 [file sj-pdf-1-dhj-10.1177_20552076261474266.pdf]

Supplementary Document 1

Database Search Strategy

PubMed Search Strategy

Search terms in Title/Abstract

(self-management OR self-control OR “social cognitive theory” OR self-efficacy OR self-regulation OR “behavio* modification” OR “behavio* change” OR “behavio* change technique” OR BCT) AND (mHealth OR “mobile health” OR “digital health” OR eHealth OR telehealth OR “digital behavio change intervention”) AND (obes* OR overweight OR weight) AND (tailor* OR personali* OR customi* OR individuali*)

Results: 236 records

Publication inclusion dates: 2015 - search date

Search date: 25. 3. 2025

Web Of Science Search Strategy

Search terms in Title

Search terms Abstract

Final search: #1 AND #2

(self-management OR self-control OR “social cognitive theory” OR self-efficacy OR self-regulation OR “behavio* modification” OR “behavio* change” OR “behavio* change technique” OR BCT) AND (mHealth OR “mobile health” OR “digital health” OR eHealth OR telehealth OR “digital behavio change intervention”) AND (obes* OR overweight OR weight) AND (tailor* OR personali* OR customi* OR individuali*)

Results: 123 records

Publication inclusion dates: 2015 - search date

Search date: 25. 3. 2025

SCOPUS Search Strategy

Search terms in Title/Abstracts/Keywords

(self-management OR self-control OR “social cognitive theory” OR self-efficacy OR self-regulation OR “behavio* modification” OR “behavio* change” OR “behavio* change technique” OR BCT) AND (mHealth OR “mobile health” OR “digital health” OR eHealth OR telehealth OR “digital behavio change intervention”) AND (obes* OR overweight OR weight) AND (tailor* OR personali* OR customi* OR individuali*)

Results: 331 records

Publication inclusion dates: 2015 - search date

Search date: 25. 3. 2025

ProQuest Search Strategy

Search terms in Title/Abstracts

(self-management OR self-control OR “social cognitive theory” OR self-efficacy OR self-regulation OR “behavio* modification” OR “behavio* change” OR “behavio* change technique” OR BCT) AND (mHealth OR “mobile health” OR “digital health” OR eHealth OR telehealth OR “digital behavio change intervention”) AND (obes* OR overweight OR weight) AND (tailor* OR personali* OR customi* OR individuali*)

Results: 93 records

Publication inclusion dates: 2015 - search date

Search date: 25. 3. 2025

CINAHL Search Strategy

Search terms in Title/Abstracts

(self-management OR self-control OR “social cognitive theory” OR self-efficacy OR self-regulation OR “behavio* modification” OR “behavio* change” OR “behavio* change technique” OR BCT) AND (mHealth OR “mobile

health" OR "digital health" OR eHealth OR telehealth OR "digital behavio change intervention") AND (obes* OR overweight OR weight) AND (tailor* OR personali* OR customi* OR individuali*)

Results: 41 records

Publication inclusion dates: 2015 - search date

Search date: 25. 3. 2025
